# Bright Light Therapy and Circadian Cycles in Institutionalized Elders

**DOI:** 10.3389/fnins.2020.00359

**Published:** 2020-05-06

**Authors:** José A. Rubiño, Antoni Gamundí, Mourad Akaarir, Francesca Canellas, Rubén Rial, M. Cristina Nicolau

**Affiliations:** ^1^Laboratori de Neurofisiologia del Son i Ritmes Biològics, Institut d’Investigació Sanitária Illes Balears (IDISBA), Universitat Illes Balears (UIB), Palma de Mallorca, Spain; ^2^Institut d’Investigació Sanitaria Illes Balears (IDISBA), Hospital Universitari Son Espases (HUSE), Palma de Mallorca, Spain

**Keywords:** aging, bright light therapy, cognitive impairment, circadian rhythms, sleep quality

## Abstract

**Background:**

Bright light therapy has been found to be an efficient method to improve the main parameters of circadian rhythms. However, institutionalized elders may suffer reduced exposure to diurnal light, which may impair their circadian rhythms, cognitive performance, and general health status.

**Objectives:**

To analyze the effects of 5 days of morning exposure for 90 min to bright light therapy (BLT) applied to institutionalized elderly subjects with mild/moderate cognitive impairment.

**Subjects:**

Thirty-seven institutionalized subjects of both sexes, aged 70–93 years.

**Methods:**

The study lasted three consecutive weeks. During the second week the subjects were submitted to BLT (7000–10,000 lux at eye level) on a daily basis. Cognition, attention, and sleep quality were evaluated at the beginning of the first and third week. Circadian variables were recorded continuously throughout the 3 weeks. Non-invasive holders and validated tests were used to analyze the variables studied.

**Results:**

After BLT we have found significant improvements in general cognitive capabilities, sleep quality and in the main parameters of the subject’s circadian rhythms. The results show that merely 90 min of BLT for five days seems to achieve a significant improvement in a constellation of circadian, sleep, health, and cognitive factors.

**Conclusion:**

Bright light therapy is an affordable, effective, fast-acting therapy for age-related disturbances, with many advantages over pharmacological alternatives. We hypothesize these effects were the result of activating the residual activity of their presumably weakened circadian system.

## Introduction

The circadian system (CS) enables environmental challenges to be predicted. The hypothalamic suprachiasmatic nucleus (SCN) is the master pacemaker, whose output oscillates over an approximate period of 24 h and drives all day-night rhythms of the organism including, in a non-exhaustive list, sleep-wake cycles, hormonal secretion, and core body temperature.

The SCN receives input from intrinsically photosensitive retinal ganglion cells (ipRGCs) to warrant synchronizing internal variables with the geophysical cycles of light and dark. Nevertheless, the SCN is not alone in regulating the CS. Indeed, additional cellular clocks can be found in every tissue. Of course, these peripheral clocks work under the command of the master SCN clock, which coordinates the temporal organization of peripheral clocks, tissues and organs. With aging, many aspects of the circadian regulation change, with reduced amplitude and phase advance (Hofman and Swaab, 2006). Alterations in the regulation of circadian rhythms are furthermore thought to contribute to the symptoms of a number of other conditions, so, risk is increased in old age, e.g., sleep disturbances and environmental depression (Weinert and Waterhouse, 2007). Elderly people also show a reduced light transmission through the eye caused by reduced pupil diameter and yellowing of the lens, which hinders light entry for rhythm synchronization (Hofman and Swaab, 2006).

Environmental light is the main controller (Zeitgeber) of human circadian rhythms (Lewy, 1983; Lewy et al., 1984; Czeisler et al., 1986, 1989) through the synthesis of melatonin by the pineal gland during periods of darkness. Melatonin gives the body time cues and is used as a marker of the circadian clock. Light during the night-time can acutely cease melatonin production and, depending on the timing of exposure, light can phase shift the timing of melatonin production. Core body temperature is also used as a marker of the circadian clock and has an inverse relationship with melatonin secretion and plasmatic levels. It reaches a peak late in the afternoon/early evening and a trough late at night/early in the morning (Gooley et al., 2010; Figueiro, 2013).

The aging process involves neurodegenerative changes: slow mental reactions, attentional deficits and memory lapses that, in the first stages, define mild cognitive impairment (MCI), an objective and measurable deficit in cognitive functions, but with preservation of daily activities. MCI may be progressively aggravated several years prior to the appearance of clinical signs and it ends in various types of dementia, including Alzheimer’s disease (AD). This preclinical phase increases in proportion to the subject’s previous intellectual capacity (Petersen et al., 2001, 2009; Rentz et al., 2001) and is shorter in subjects with borderline cognitive capabilities (Silveri et al., 2007). Indeed, MCI is thought to represent a transitional state between normal aging and early AD (Grundman et al., 2004; Tomaszewski Farias et al., 2009).

Circadian rhythm disturbances are an important factor in the production of MCI (Tranah et al., 2011). Indeed, disruption of the CS affects cognitive levels, altering the memory and capability of information processing in the elderly (Oosterman et al., 2009). In particular, an improper pattern of light exposure caused by impaired vision (Lockley et al., 2007), reductions in the phase-delaying response to moderate light levels (Duffy et al., 2007), circadian phase misalignment (Dijk et al., 1999; Yoon et al., 2003), as well as reduced exposure to diurnal and/or increased exposure to nocturnal levels of light, respectively (Campbell et al., 1988; Mclntyre et al., 1989). These may be important factors contributing to cognitive deficits and the conversion of MCI into AD (Riemersma-van der lek et al., 2008; Cardinali et al., 2010), and these factors frequently occur in institutionalized elderly people or when elderly people suffer mobility impairments in their own home (Ancoli-Israel et al., 1997; Anderiesen et al., 2014). In parallel with these disturbances, the synthesis, secretion, and plasma levels of melatonin undergo significant age-related reduction (Scholtens et al., 2016). These disturbances are aggravated in elderly people suffering from insufficient exposure to diurnal light, as well as in patients in preclinical and clinical AD stages (Skene and Swaab, 2003; Wu et al., 2003, 2007; Wu and Swaab, 2005).

Bright light therapy (BLT) applied during the day, is an effective mechanism to diminish the alterations of circadian rhythms in the elderly (Touitou and Haus, 2000). BLT increases sleep quality, sleep stability, and sleep efficiency (proportion of bed rest without sleep); and decreases the number of night awakenings and daytime sleepiness. Moreover, BLT improves mood and cognitive capabilities and decreases agitation and hallucinations (Ancoli-Israel et al., 2003; Dowling et al., 2008; Riemersma-van der lek et al., 2008). BLT is beginning to be recognized as an affordable, effective, fast-acting therapy with many advantages over traditional pharmacological alternatives (Sloane et al., 2008).

The present report aims to analyze, using non-invasive recording methods, the beneficial effects of BLT on physiological and psychological performance of institutionalized elderly with mild/moderate cognitive impairment. We show that BLT, applied in temporal coherence with the general circadian organization, counteracts many signs of cognitive and behavioral decline that are often observed in the elderly.

## Materials and Methods

### Participants

Participants were elderly, living in a nursing home on the island of Mallorca (latitude: ∼40°N). The study was conducted over three consecutive weeks, between April and May (spring) with an average of 60% sunny days, 14°C (night) and 21°C (day) and 13 h 12 min of natural light. A total of 37 subjects of both sexes (33 women and 4 men) were studied. All participants and caregivers received complete information regarding the purpose and characteristics of the study and signed an informed consent form before being included in the study. Neither subjects nor caregivers received financial compensation for their participation in the study.

All procedures were carried out under authorization of the Research Ethics Committee of the Government of the Balearic Islands (IB/1409/10 PI).

Inclusion criteria were mild/moderate cognitive impairment (Mini Mental State Exam, MMSE, between 25 and 19), good mobility and with conserved visual and auditory acuity. The mean age of the sample was 79.9 years (range 70–93) and the educational average was 6.8 school years. The usual pharmacological treatment was maintained, but participants with medication that presumably exerted effects on the CS were excluded. 23 out of 37 subjects had concomitant medical conditions: high arterial pressure, anxiety, insulin-dependent diabetes, dyslipidemia, osteoporosis, osteoarthrosis, cardiac arrhythmia, and chronic pain.

All participants were free to move through the different spaces of the institution, with the possibility of remaining outdoors. Participants and caregivers were instructed to complete a sleep agenda with information about the onset and offset of sleep, as well as the time and duration of occasional naps. However, the reliability of the resulting agenda was quite low, so the results were not used.

### Study Design

The effects of BLT (independent variable) were studied on cognitive, functional and attentional status, on sleep quality and on the circadian rhythm of activity and wrist temperature (WT) (dependent variables), for 3 weeks (5 days/week).

This study had a quasi-experimental design with pre-test and post-test measures.

After explaining the objectives and the experimental procedures, participants signed the written consent and non-invasive described variables were evaluated.

### Light Exposure Recording

**FIGURE 1:**
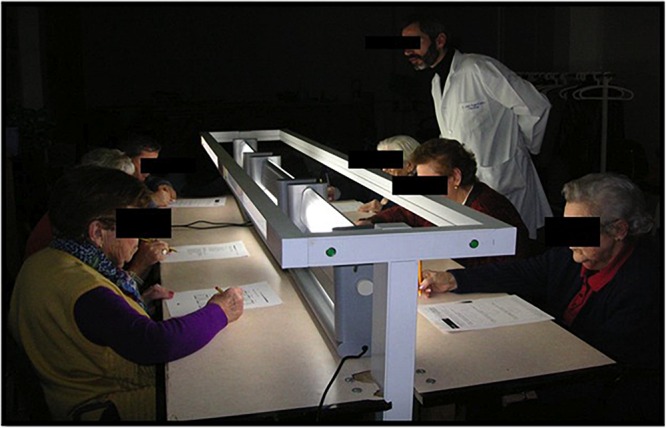
Design of work table for exposition to bright light therapy (OSAL-LUM^®^ polyvalent white light emitters).

**FIGURE 2:**
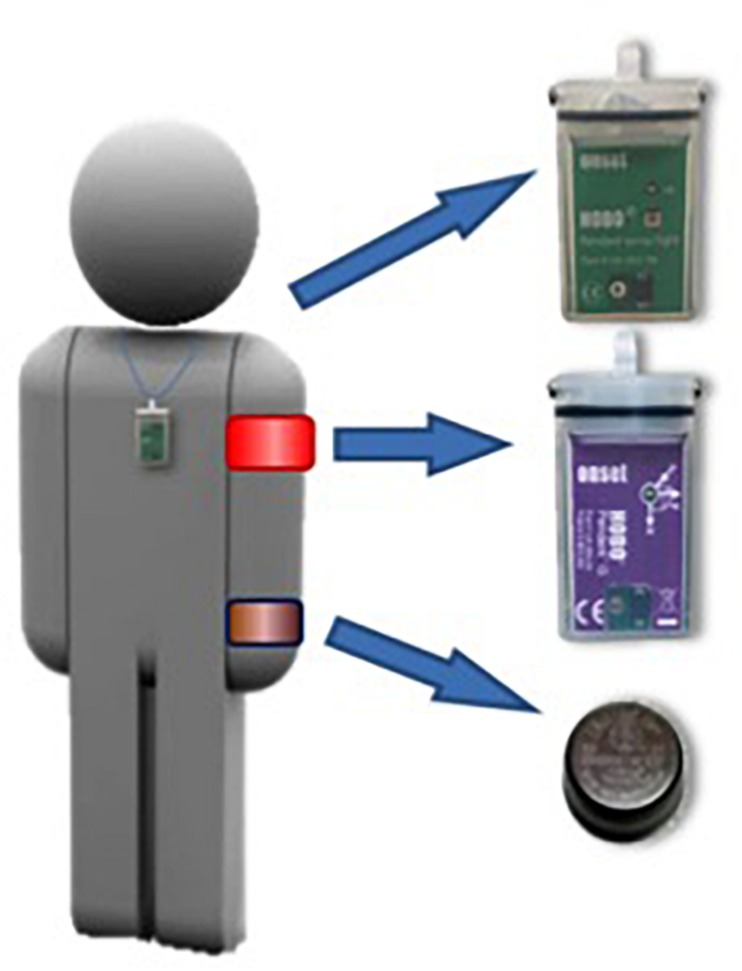
Placement of sensors for recording exposure to light, peripheral temperature, and activity.

During 3 weeks the participants were seated between 10:30 and 12:00 a.m. (90 min) at a work table with two rows of seats (total six participants/session) performing their routine daily tasks – reading, writing, knitting, and social activities – while exposed to the habitual light levels of the institution during weeks 1 and 3. During week 2, between 10:30 and 12:00 a.m., the participants were exposed, in the same table, to two rows of three fluorescent lights (OSAL-LUM polyvalent white light emitters, Model 255HFDIM, Yanche. S.L.), totaling six lights, 68 cm long that provided, 7000–10,000 lux, 350–750 nm, at an approximate distance of 40–60 cm from the eyes of every participant (Figure 1). The period of BLT was selected aiming at minimal interference with the daily activities of the subjects. Light exposure was recorded using individual Light Data Loggers (UA-002-64, Onset Computer, Bourne, MA, United States) (Figure 2) with capacity to record between 0 and 320,000 lux (Martínez-Nicolás et al., 2013). The data loggers were used along the 3 weeks as neck pendants during waking hours and placed on the bedside table during sleeping hours. They were programmed to store the light intensity (lux) impinging the eyes of the subjects every 10 min over the three experimental weeks. The reliability of the loggers was contrasted with a lux-meter (Sekonic, model 246, Tokio, Japan) and the discrepancies between different data holders and the calibrating illuminance meter calibrated in lux were, in every case, lower than ±5%.

The period of BLT was selected between 10:30 and 12:00 a.m., after breakfast, for minimal interference with the daily routine of the participants.

### Sleep Quality Test

Subjects’ sleep quality was evaluated using the Oviedo Sleep Questionnaire (OSQ) (García et al., 2000), a semi-structured interview to quantify sleep satisfaction (score: 1 low, 7 max), insomnia (score: 9 low, 45 max), and hypersomnia (score: 3 low, 15 max). This test was applied at the beginning of week 1 and at the end of week 3 between 10:00 and 12:00 a.m.

### Evaluation of Cognitive Variables and Attention

Cognitive, functional and health variables were assessed using the tests described in Table 1.

**TABLE 1:** Evaluation of cognitive and functional variables.

**Cognitive state**	**MMSE. Spanish validation (Blesa et al., 2001)**
Memory	Memory Scale, with subtests for immediate, delayed and working memory, word learning, and word recognition. Spanish validation (Taussig et al., 1992).
Functional state	Global Deterioration Scale. Spanish validation (Peña-Casanova et al., 2009).
Sustained attention	TMT-A. Spanish validation (Llinàs-Reglà et al., 2017).
Selective attention	Comprehensive Trail Making Test, CTMT-3. Spanish validation (del Ser Quijano et al., 2004).
Divided attention	Stroop. Spanish validation (Peña-Casanova et al., 2009).

### Activity and Wrist Skin-Temperature Recording

Activity and wrist skin-temperature can be used as markers of the rhythms to assess the function of the CS (Hofstra and de Weerd, 2008). Measuring the rest-activity rhythm by actigraphy is a simple, non-invasive method and also serves for indirect evaluation of the sleep-wake cycle.

Subjects’s activity was continuously monitored using activity loggers (Hobo G Acceleration Data Logger, MA, United States) placed over the non-dominant arm by means of a sports band, with its X-axis parallel to the humeral bone (Ortiz-Tudela et al., 2010; Figure 2). These meters recorded the combined X, Y, and Z-axis accelerations. The sensors were programmed to average the vector activity every 30 s.

Wrist temperature constitutes a good, reliable and minimally invasive method for evaluating the circadian rhythm of body temperature (Sarabia et al., 2008; Ortiz-Tudela et al., 2010). Peripheral WT was sampled every 10 min using miniature temperature holders (Thermochron iButton DS1921H, Dallas, TX, United States, maximum sensitivity: 0.1°C) attached to a cotton sports bracelet with the sensor surface placed on the inside of the wrist over the radial artery of the non-dominant arm (Figure 1).

### Circadian Analysis

All circadian parameters were submitted to circadian analysis using Circadianware Software (University of Murcia, Spain). To eliminate artifacts, periods of zero activity were rejected. In addition, data showing more than 3 SD (±3 SD covers 99.7% of normally distributed data) were eliminated (Ortiz-Tudela et al., 2010). As some subjects returned home on weekends, their daily routine changed. In these cases, the weekend data were also deleted. Light intensities were sampled every 10 min and the results were averaged for waking and sleeping periods. Motor activity was expressed in degrees of the vector’s position change per minute by dividing the original values 10 times to obtain the averaged activity in periods of 10 min. WT was expressed in degrees Celsius. Results were expressed as the week average of each subject’s WT and motor activity.

Wrist temperature and activity were analyzed using parametric tests (Cosinor and Rayleigh tests). The Cosinor adjustment fits the data to the best 24 h sinusoidal regression line and provides the mesor, acrophase, and amplitude of the rhythm. The mesor is the average value around which the variable oscillates. The acrophase is the time of day at which the highest point of the fitted-cosine curve occurs. The amplitude is the difference between the mesor and the peak of the waveform function fitted to the data (Díez-Noguera, 2006; Haffen, 2009). The Rayleigh test (RAY) is derived from the Cosinor analysis and enables the rhythm’s phase stability to be calculated on successive days. It provides a vector (R) placed in the center of a circular clock (24 h) whose angle indicates the mean peak time (acrophase) of each subject during every experimental week, together with the time of maximal amplitude and the confidence limits in amplitude and time. The length of the vector (between 0 and 1) is proportional to the amplitude of the acrophase. Although many circadian rhythms in humans and WT are not sinusoidal, the analysis assumes sinusoidal regression. Non-parametric analyses were also performed in order to calculate additional variables. The non-parametric variables are sensitive indices of disturbances in the rest-activity rhythm. They are also sensitive to the effect of treatment in elderly in whom degeneration of the SCN is present, even with a very small sample size (Van Someren et al., 1999; Carvalho-Bos et al., 2007):

*IS* (inter-day stability) quantified the regularity or consistency of the rhythm pattern and varied between 0 for Gaussian noise and 1 for perfect stability.
*IV* (intra-day variability) quantified the fragmentation of the rhythm and varied between 0 when the wave was sinusoidal and 2 for Gaussian noise.
*RA* (relative amplitude) calculated as the average difference between M5 (5 consecutive hours of maximum values) and L10 (10 consecutive hours of minimum values), divided by the sum of M5 and L10 for WT, as well as the difference between M10 (10 h maximum values) and L5 (5 h minimum values) divided by the sum of M10 and L5 for motor activity (Ortiz-Tudela et al., 2010).
*CFI* (Circadian Function Index) calculated from the average of the three variables, IS, IV, and RA. The IV original values were reversed prior to CFI calculation. The CFI ranged from 0 (absence of circadian rhythmicity) to 1 (a robust circadian rhythm). The CFI is a powerful method to assess CS status with a specific accuracy and to differentiating activity from rest (Ortiz-Tudela et al., 2010).

For statistical analysis the IBM SPSS statistics package, V 20.1 was used. Student’s *t*-test was used for correlated samples to establish the difference between pre- and post-BLT tests. Variance analysis with repeated measures, followed by Bonferroni *post hoc* tests were used to discriminate between weeks and treatments.

## Results

### Light Exposure

**FIGURE 3:**
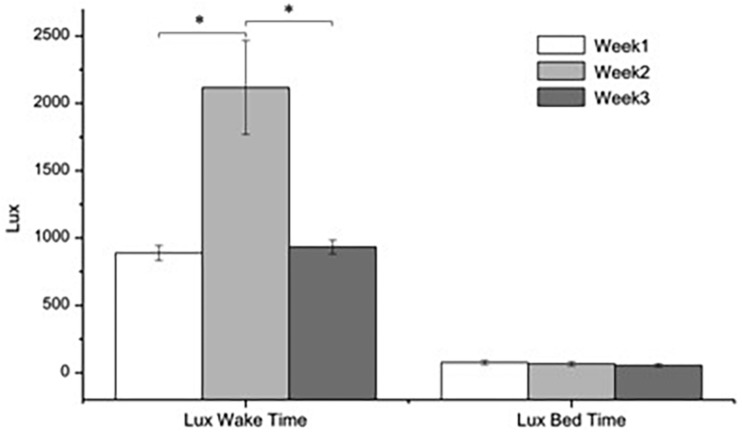
Average levels (±SEM) of light recorded 10 min by the subject’s HOBO pendants. ANOVA two-factors, (*F*_(2,35)_: *p* < 0.001).

Figure 3 shows the week-average levels of light recorded every 10 min during wake time (7:00 to 20:50) and sleep time (21:00 to 6:50) in accordance with the institution time schedule. Comparing the amount of light recorded in week 2 (with BLT) with those of weeks 1 and 3 (without BLT) the difference was highly significant (*F*_2_,_35_ = 11.59, *p* < 0.001). Conversely, the difference between the 3 weeks during sleeping time was non-significant (*F*_2_,_35_ = 0.48, ns).

The light levels recorded in the different rooms of the institution during weeks 1 and 3 were similar to those recorded by the subject’s pendants in the same intervals, so were not represented.

### Effects of BLT on Sleep

The results of the Oviedo Questionnaire (Table 2) show that sleep satisfaction improved after BLT and nocturnal insomnia was reduced. Diurnal hypersomnia, however, remained without change.

**TABLE 2:** Results of the *t*-test applied to the Oviedo Sleep Questionnaire.

**Variables**	**Pre-BLT**	**Post-BLT**	**Significance**
Sleep satisfaction	4.03 ± 0.02	4.51 ± 0.04	**0.04**
Nocturnal insomnia	20.73 ± 0.68	15.70 ± 1.3	**0.007**
Diurnal hypersomnia	6.75 ± 0.06	6.0 ± 0.04	0.153 ns

*All indexes are expressed as mean ± SD. Significant differences are marked in bold. BLT, bright light therapy; ns, non-significant.*

### Effects of BLT on Cognitive, Functional and Attention Variables

The results of tests used to record changes in cognitive, functional and attention states are shown in Table 3.

**TABLE 3:** Results of the Student’s *t*-test applied to the memory attention and global deterioration.

	**Pre-BLT**	**Post-BLT**	**Significance**
Cognitive state	22.72 ± 6.53	24 ± 5.92	***p* < 0.001**
Immediate memory	3.5 ± 1.50	4.84 ± 1.89	***p* < 0.001**
Delayed memory	4.41 ± 3.55	5.33 ± 2.74	***p* < 0.05**
Working memory	2.25 ± 0.86	2.66 ± 0.88	***p* < 0.01**
Learning	1.08 ± 2.39	0.50 ± 1.56	*p* = 0.759 ns
Recognition	20.58 ± 2.96	21.33 ± 2.70	***p* < 0.05**
Sustained attention	2.71 ± 0.05	2.38 ± 0.02	*p* = 0.08 ns
Selective attention	3.49 ± 0.08	3.13 ± 0.19	*p* = 0.074 ns
Divided attention	3.79 ± 0.60	8.69 ± 0.77	***p* < 0.05**
Functional state	3.10 ± 1.26	2.72 ± 5.92	***p* < 0.001**

*All indexes are expressed as mean ± SD. Significant differences are marked in bold. BLT, bright light therapy; ns, non-significant.*

After BLT, the cognitive state and the immediate, delayed and working memory were improved. Small but significant improvements also appeared in word recognition, global deterioration (GD) and in divided attention, although no-significant differences were found neither in learning capability nor in sustained and selective attention.

### BLT and Circadian Rhythms of Temperature and Activity

After BLT, the WT mesor and amplitude of the rhythm were increased (Table 4). These effects continued to be significant in week 3. In addition, BLT delayed the acrophase when weeks 1 and 2 and weeks 1 and 3 were compared. Notably, no significant difference was found between weeks 2 and 3. IS and relative amplitude showed significant improvements. CFI showed delayed improvements, with significant differences between weeks 1 and 3. Finally, the RAY evidenced significant improvements in weeks 2 and 3.

**TABLE 4:** Results of the ANOVA analysis applied to the mean WT circadian parameters over the 3 weeks of the study.

**Variables**	**Week 1**	**Week 2**	**Week 3**	**ANOVA *F*_(1_._107)_**	***p* between weeks (Bonferroni *post hoc***)		
					1–2	1–3	2–3
Wrist temperature							
Mesor	32.81 ± 0.12	34.24 ± 0.15	33.17 ± 1.53	*F* = 7.20, *p* = 0.001	**0.021**	0.042	**0.039**
Amplitude	1.03 ± 0.05	1.32 ± 0.06	1.47 ± 0.08	*F* = 6.08, *p* = 0.003	**0.008**	**0.038**	**0.034**
Acrophase	23:40 ± 16min	01:10 ± 10*min*	01:31 ± 10*min*	*F* = 17.78, *p* = 0.001	**0.001**	**0.001**	0.814
IS	0.47 ± 0.03	0.55 ± 0.03	0.62 ± 0.02	*F* = 7.35, *p* = 0.001	**0.002**	**0.002**	0.642
IV	0.16 ± 0.02	0.12 ± 0.01	0.13 ± 0.01	*F* = 1.58, *p* = 0.21 ns			
RA	0.02 ± 0.01	0.03 ± 0.01	0.05 ± 0.01	*F* = 16.5, *p* = 0.001	0.078	**0.001**	**0.002**
RAY	0.83 ± 0.01	0.92 ± 0.01	0.88 ± 0.01	*F* = 8.43, *p* = 0.001	**0.006**	0.634	**0.026**
CFI	0.49 ± 0.00	0.49 ± 0.01	0.53 ± 0.01	*F* = 7.58, *p* = 0.001	0.04	**0.305**	0.003

*All indexes are expressed as mean ± SD (Standard Deviation). Significant differences between weeks are marked in bold. IS, inter-day stability; IV, intra-day variability; RA, relative amplitude; RAY, Rayleigh tests; CFI, Circadian Function Index; ns, non-significant.*

In relation to activity, IS and CFI remained without significant changes. In contrast, IV was reduced after BLT in week 2, while the relative amplitude as well as the Rayleigh coefficient increased. However, the effects disappeared in week 3 (Table 5).

**TABLE 5:** Results of the ANOVA analysis applied to the mean activity parameters over the 3 weeks of the study.

**Variables**	**Week 1**	**Week 2**	**Week 3**	**ANOVA *F*_(1.107)_**	***p* between weeks (Bonferroni *post hoc***)		
					1–2	1–3	2–3
Activity							
Mesor	13.48 ± 0.66	15.64 ± 0.63	15.14 ± 0.39	*F* = 3.84, *p* = 0.02	0.024	0.019	**0.81**
Amplitude	7.31 ± 0.33	9.03 ± 0.32	9.13 ± 0.28	*F* = 10.57, *p* = 0.001	**0.016**	**0.001**	0.63
Acrophase	13:48 ± 10min	14:08 ± 8min	14:27 ± 9min	*F* = 7.75, *p* = 0.001	0.059	**0.001**	0.245
IS	0.30 ± 0.05	0.33 ± 0.01	0.32 ± 0.006	*F* = 0.94, *p* = 0.39 ns			
IV	0.99 ± 0.01	0.87 ± 0.02	0.98 ± 0.01	*F* = 9.68, *p* = 0.001	**0.042**	0.623	**0.038**
RA	0.73 ± 0.02	0.84 ± 0.02	0.77 ± 0.01	*F* = 4.10, *p* = 0.019	**0.010**	0.39	**0.028**
RAY	0.80 ± 0.03	0.89 ± 0.02	0.85 ± 0.01	*F* = 3.09, *p* = 0.049	**0.003**	**0.046**	0.53
CFI	0.51 ± 0.01	0.53 ± 0.01	0.51 ± 0.01	*F* = 0.41, *p* = 0.65 ns			

*All indexes are expressed as mean ± SD (Standard Deviation). Significant differences between weeks are marked in bold. IS, inter-day stability; IV, intra-day variability; RA, relative amplitude; RAY, Rayleigh tests; CFI, Circadian Function Index; ns, non-significant.*

## Discussion

### Light

The elderly in their eighth and ninth decades retain only 10% of a 10-year-old child’s circadian photoreception, mostly due to opacification of the lens, reductions in pupil diameter and loss of photoreceptor sensitivity (Yang et al., 2002; Charman and Chateau, 2003; Turner and Mainster, 2008), causing deficits in the absorption of 400–500 nm visible light, which is essential for circadian regulation (Najjar et al., 2014). Several studies of BLT proposed the use of 10,000 lux at eye level to improve cognitive parameters, sleep quality and circadian functions in the elderly (Mishima et al., 1994; Campbell et al., 1995a,b,c; Remé et al., 1996; Sloane et al., 2008; Brouwer et al., 2017). These light levels are considered safe (Terman and Terman, 2005). Meanwhile, the DELTA Portfolio (Lighting Research Center, 1999) recommends up to 3200 lux at eye level for institutionalized elderly, i.e., sevenfold higher than the 500 lux recommended by the European Standard (EN12464-1).

The available light levels perceived by the subjects during daytime were discrete in absolute and relative terms (Figure 2) and well below the European Standard. Conversely, the values of light during night-time were much higher (40 lux/10 min), because of the institutional established routines, in contrast with the recommended 5 lux levels. Most likely, the exposure to light during sleep exacerbates the already diminished age-related levels of melatonin (Wu et al., 2003, 2007; Wu and Swaab, 2005) and reduces diurnal-nocturnal light contrast (Rubiño et al., 2017). Consequently, the subjects selected were ideally suited to study the effect of BLT, which, applied during morning time, improves the activity of the SCN clock, which is extremely sensitive to deficits in morning light (Minors et al., 1991; Rüger et al., 2013), and improving the cognitive, functional and clinical variables (Mishima et al., 1994; Dowling et al., 2008). Accordingly, we decided to use 10,000 lux light sources with high intensity within the optimal range for the human CS (464–484 nm) (Berson et al., 2002; Rea et al., 2002; Weng et al., 2009; Reiter et al., 2011) and the maximal capacity for improving nocturnal secretion of melatonin in elderly (Sörensen and Brunnström, 1995; Murphy and Campbell, 1996; Khalsa et al., 2003).

### Cognitive Variables

We used the Spanish validated version (Blesa et al., 2001) of the MMSE test that is highly sensitive for the detection of MCI (Folstein et al., 1975) and to classify the mental status (Flicker et al., 1991). It is often used to evaluate mental decline in institutionalized elderly patients (Reisberg et al., 1984, 1985; Franssen et al., 1991). It has been often used to analyze temporal and spatial orientation, immediate and delayed memory, calculation capacity and verbal communication (Ito et al., 1999; Yamadera et al., 2000; Graf et al., 2001; Hickman et al., 2007; Cochrane et al., 2012).

We observed discrepancies in the cut-off scores to define the MCI (Reisberg et al., 1985; Franssen et al., 1991; Tombaugh and McIntyre, 1992; Crum et al., 1993; Kukull et al., 1994; Mitchell and Shiri Feshki, 2009). Consequently, we used the score of ≤25, a rather high figure (Blesa et al., 2001), to mark the frontier between normality and mild impairment in order to increase the screening sensitivity of BLT. We complemented the MMSE with the Global Deterioration Scale (GDS) (Peña-Casanova et al., 2009), finding that the application of BLT caused significant increases in the MMSE score (+1.2 points) and decreases in the GDS (Table 3). So, despite the short exposure to BLT we observed significant improvements in cognition agreeing with previous reports (Ito et al., 1999; Graf et al., 2001; Riemersma-van der lek et al., 2008; Burns et al., 2009).

The Wechsler Memory Scale is used to record memory impairment (Taussig et al., 1992). BLT caused significant improvements in all subtests, with the single exception of the separate subtest word learning in which the effect was non-significant (Table 3).

The effects of BLT on attention levels were analyzed using the Trail Making Test (TMT) (del Ser Quijano et al., 2004; Llinàs-Reglà et al., 2017) that assesses visual search speed and sustained attention. We also used the Comprehensive TMT 3, to analyze selective attention. However, BLT caused non-significant changes neither for sustained nor selective attention. By contrast, divided attention was highly improved, from a score of 3.8 to 8.7 (Table 3). Interestingly, divided attention is the most complex attentional level, which would, most likely, have been the most deteriorated one.

### Sleep

In institutionalized elderly with dementia, cognitive deterioration is often accompanied by sleep disturbances (Pollak and Perlick, 1991), with further deteriorations in their capacity for daily activities, increasing the need for care (Llinàs-Reglà et al., 2017). The main sleep disturbances in the elderly are nocturnal insomnia, excessive daytime sleepiness and diminished sleep quality (Bobes et al., 1998; García et al., 2000).

Subjects showed poor sleep quality (Table 2), with their scores oscillating around 50% of the optimal for sleep satisfaction, nocturnal insomnia, and diurnal hypersomnia. In all likelihood, these results were due to the extremely long duration of bedtime that was established as a norm in the institution (from 21.00 to 07.00, i.e., 600 min of total bed time), hugely contrasting with the 360 min of total sleep time reported for healthy, 60–70 year-old subjects (Wilckens et al., 2014; Kurina et al., 2015). Considering a normal sleep efficiency of 77.5% (Dijk et al., 1999) for elderly people, the total bedtime for healthy elderly people should range around ∼465 min. First, the excessive bedtime could have decreased the sleep drive. Second, the impaired sleep continuity and depth could explain the cognitive impairments (Wilckens et al., 2014). In the same vein, the frequent awakenings caused by switching-on the lights for diapers check and removal must have impaired the normal age-related reductions in melatonin secretion even more. Nevertheless, we found that after BLT subjects showed significant improvements in sleep satisfaction and nocturnal insomnia. The change, however, was not significant for daytime hypersomnia. Notably, previous reports of applying this questionnaire to elderly people observed similar sleep satisfaction and diurnal insomnia indexes (Rüger et al., 2013).

### Circadian Rhythms

The circadian rhythm of peripheral WT, exhibits an inverse phase relationship with core temperature and represents a non-invasive, robust, and easy to record index of the circadian rhythms (Ortiz-Tudela et al., 2010; Martínez-Nicolás et al., 2013). Because WT increases during rest periods (night) and decreases during periods of activity (day), it has been suggested that drowsiness may be directly related to WT and not to core temperature (Van Someren, 2004; Kräuchi et al., 2005).

Bright light therapy seemed to cause immediate effects in most parameters of the WT rhythm, and in many cases, the improvements appeared and/or persisted in week 3, suggestive of delayed post-effects modifying the settings of the circadian clock. Indeed, mesor, amplitude, IS, IV, Rayleigh coefficient, and CFI were significantly improved. Moreover, BLT delayed the acrophase (Table 4). BLT may have caused similar improvements in the rhythm of activity except for IS and the CFI, in which the changes were non-significant (Table 5).

Nevertheless, BLT caused significant improvements in the rhythms of body temperature and activity, counteracting the typical clock advance observed in elderly people (Iguchi et al., 1982; Skene and Swaab, 2003; Hofman and Swaab, 2006), thereby contributing to “rejuvenating” the CS. It is noteworthy that most improvements were still significant one week after the application of BLT. Thus, BLT seemed to reset the SCN clock. In contrast, the changes in activity rhythm were non-persistent.

Although the period of the circadian rhythms depends on geophysical cycles, it is also sensitive to external masking factors that add or modify the effects of light, reinforcing or blunting the efficiency of the clock (Weinert and Waterhouse, 2007; Martínez-Nicolás et al., 2013). Consequently, the low day-night difference in light exposure before the application of BLT might have contributed to blunt the temperature and activity rhythms of the subjects, a possibility that was aggravated for the described visual impairments and their presumably weak CS. Nevertheless, BLT may have activated the residual activity of the inmates’ internal clock, thereby explaining the significant improvements in the rhythms of temperature and activity.

Low values of light were found during wake time and excessive light levels during sleep time. Therefore, the daily exposure to 10,000 lux for only 90 min could well have represented an important change, responsible for the observed constellation of circadian, sleep, well-being, and cognitive improvements and counteracting, at least in part, the low values of light found during wake time and the excessive levels recorded during sleep time. These results agree with previous ones and may confirm that BLT improves waking and arousal levels (Campbell et al., 1995b), sleep quality (Campbell et al., 1995c), and age-related cognitive disturbances (Van Someren et al., 1999; Fetveit et al., 2003; Sateia and Buysse, 2016).

### Limitations of the Study

Studies with humans involve several difficulties; in this case the most important limiting factor was the low number of participants who fulfilled the inclusion criteria for the study. Also, the inclusion of a daily agenda for every participant would greatly increase the value of the study. Nevertheless, our results showed high internal coherence and confirmed similar results obtained in other studies. Therefore, we should conclude that, despite the hindrances, the present study is demonstrative of the exquisite sensitivity of the CS of the elderly and of the possibilities of significant improvements in their quality of life.

### Future Directions

Future research is needed to evaluate the optimal light intensity, spectral distribution, etc., for improving CS functioning, sleep quality, cognitive performance, and well-being in the institutionalized elderly.

In addition, future studies should evaluate the benefits of introducing circadian-adapted ambient lighting to institutions for the elderly. New technological development in ambient lighting may help to improve the feasibility of light therapy in the new building nursing homes.

## Conclusion

In short, improper illumination – too low during waking hours and excessive during sleeping hours – may play a role in the poor sleep quality and impairments in cognition often appearing in the elderly with mild to moderate cognitive impairment, meanwhile, the present results add evidence regarding the benefits of BLT on circadian organization, sleep quality and cognitive capabilities. Thus, the present results stress the importance of illumination in institutions for elderly. Indeed, it is increasingly recognized that light is an affordable, effective, fast-acting therapeutic tool (Wirz-Justice et al., 2004; Erren and Reiter, 2009; Canellas et al., 2016).

## Data Availability Statement

The datasets generated for this study are available on request to the corresponding author.

## Ethics Statement

The studies involving human participants were reviewed and approved by the Research Ethics Committee of the Government of the Balearic Islands (IB/1409/10 PI). The patients/participants provided their written informed consent to participate in this study.

## Author Contributions

All authors conceived and contributed to design the experiments and wrote the manuscript and approved it in its final form.

## Conflict of Interest

The authors declare that the research was conducted in the absence of any commercial or financial relationships that could be construed as a potential conflict of interest.
